# Micropropagation of the Endemic *Stachys pinardii* Boiss. and Comparative Investigation of the Phytochemical Composition of Cultured and Wild-Grown Plants

**DOI:** 10.3390/molecules31203773

**Published:** 2026-10-21

**Authors:** Hatice Üstüner

**Affiliations:** Department of Biology, Faculty of Science, Akdeniz University, Antalya 07058, Turkey; h.ustuner1977@gmail.com

**Keywords:** *Stachys pinardii*, ex situ conservation, plant growth regulators, GC–MS/GC–FID metabolite profiling, specialized metabolites, nerolidol

## Abstract

This study aimed to establish an efficient micropropagation protocol for the endemic plant *Stachys pinardii* Boiss. and to characterize differences in specialized metabolite profiles between in vitro-derived and wild plants under the tested conditions. Nodal explants were cultured on Murashige and Skoog (MS) medium supplemented with different cytokinin (BAP, KIN) and auxin (IAA, NAA) combinations, with morphogenetic data analyzed by one-way ANOVA followed by Duncan’s Multiple Range Test and Tukey’s HSD test (*n* = 30 per treatment); air-dried aerial parts (upper shoots) of in vitro-derived and wild-grown plants were compared phytochemically using GC–MS for compound identification and GC–FID for relative quantification based on peak-area normalization (*n* = 3 repeated analyses of each extract). The BAP (1.0 mg/L) + IAA (2.0 mg/L) combination produced both the highest shoot number (11.43 ± 0.20 shoots per explant) and the greatest shoot length (3.36 ± 0.05 cm) among the tested treatments. GC–MS identification and GC–FID relative quantification revealed a marked difference in phytochemical composition: the relative percentage of nerolidol was 45.4% in the optimized in vitro treatment, compared with 10.3% in wild-grown plants, while, in the hexane extracts, carvacrol showed its highest relative percentage in wild-grown plants (12.0%) and was not detected in the optimized in vitro treatment. These findings indicate that in vitro culture systems can serve not only as a tool for ex situ conservation but also as a controlled platform for characterizing differences in specialized metabolite profiles under defined culture conditions, providing a basis for further investigation into the biotechnological potential of *S. pinardii*.

## 1. Introduction

Higher plants have long played an important role as sources of therapeutic agents in traditional medicine [1]. The genus *Stachys* (Lamiaceae), widely distributed in the Mediterranean and Southwest Asia [2,3], has gained significant attention due to its diverse biological activities and rich phytochemical composition [4,5,6].

**Figure 1:**
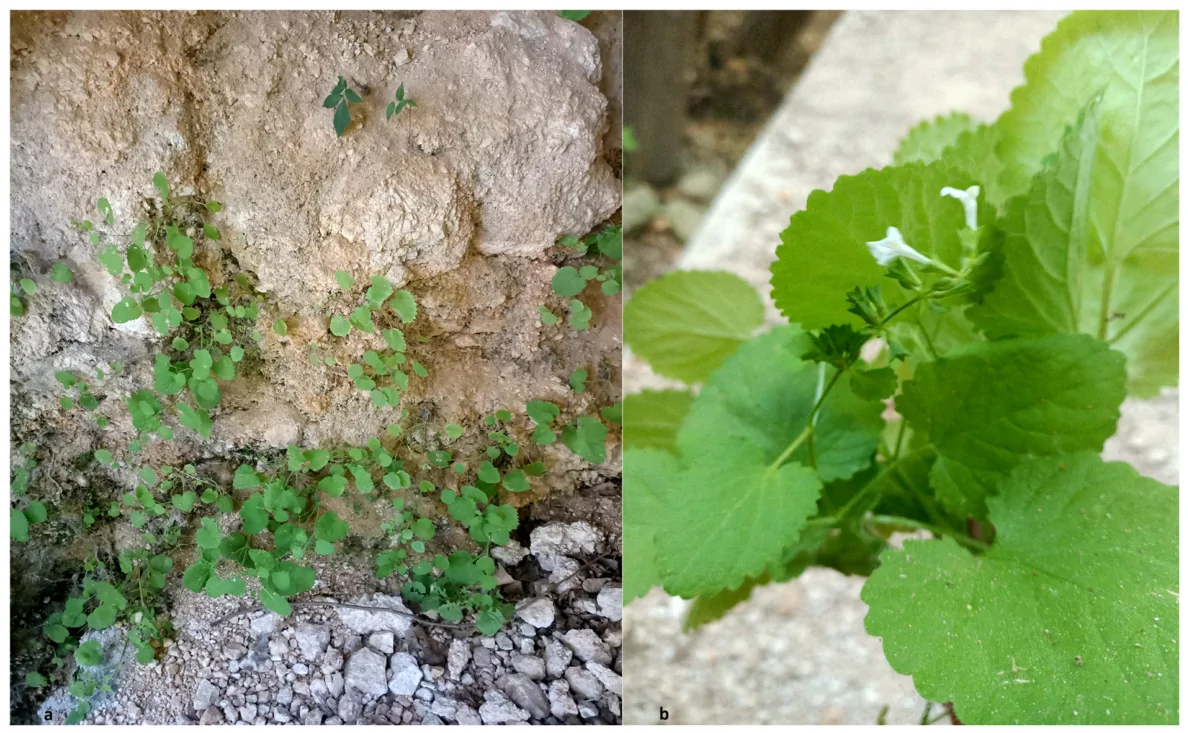
*Stachys pinardii*: (**a**) natural habitat and morphological appearance in Geyikbayırı, Antalya, Turkey. (**b**) detail of the pendulous inflorescence stems and white corolla.

*Stachys pinardii* Boiss., locally known as ‘Gevrek çayçe’, is a narrowly distributed endemic species of Turkey that grows exclusively on limestone cliffs in the Antalya region [7,8,9] (Figure 1). The species has also been reported under the former IUCN category LR(cd) (Lower Risk, Conservation Dependent), further emphasizing the relevance of conservation-oriented propagation approaches [10]. Its narrow geographic distribution and habitat specificity may increase its vulnerability to local habitat disturbance, highlighting the importance of ex situ propagation approaches that can reduce reliance on wild populations. In this context, in vitro propagation provides a valuable approach for the conservation and sustainable propagation of medicinal plants with limited natural populations [11,12]. Micropropagation has been widely investigated within Lamiaceae, including species of *Ocimum*, *Salvia*, and *Lamium*, demonstrating the applicability of in vitro culture for clonal propagation and conservation of medicinal taxa [11,12,13,14,15,16]. More specifically, an in vitro micropropagation protocol has recently been reported for the threatened endemic *Stachys cretica* subsp. *kutahyensis*, further supporting the relevance of tissue culture approaches within the genus *Stachys* [17]. Phytochemical investigations of the genus *Stachys* have revealed a diverse range of specialized metabolites (traditionally referred to as secondary metabolites), including volatile constituents, and considerable variation in chemical composition among species and taxa [4,5,6,18,19]. 

Several Stachys taxa in Turkey have documented ethnobotanical uses and are commonly consumed as herbal tea, with reported applications for various ailments [20]. However, specific ethnobotanical documentation concerning the form and extent of traditional medicinal use of *S. pinardii* appears to be limited.

Previous studies on *S. pinardii* have demonstrated its phytochemical and biological relevance through characterization of its volatile constituents and total phenolic content [19], as well as its essential oil composition and associated anticancer, antidiabetic, and antioxidant activities [9]. However, previous studies have focused on the phytochemical composition and biological activities of wild-collected material, whereas, to our knowledge, no study has established a micropropagation system for *S. pinardii* and directly compared the phytochemical profiles of clonally derived in vitro material with those of the corresponding wild-grown plants. This knowledge gap provided the rationale for selecting *S. pinardii* for the present comparative study. Among the constituents previously reported for *S. pinardii*, nerolidol is of particular interest because of its diverse pharmacological and biological properties [9,21].

Beyond their value for ex situ conservation, controlled in vitro culture systems provide an experimental platform for investigating differences in specialized metabolism and phytochemical composition under defined culture conditions. In vitro culture systems and related physiological conditions can be associated with changes in specialized metabolism and phytochemical composition [22,23,24]. Therefore, a comparative phytochemical evaluation between wild and in vitro-derived plants is important for characterizing these shifts and assessing the phytochemical characteristics of the micropropagated material.

The rationale for combining micropropagation with comparative phytochemical profiling lies in the potential of controlled in vitro environments and plant growth regulator (PGR) treatments to support ex situ conservation while providing a controlled system for investigating differences in phytochemical profiles. Consequently, the primary objective of this research was to establish an efficient regeneration and rooting protocol using nodal explants for clonal propagation, thereby minimizing genotypic variation during comparative phytochemical analyses. A further objective was to evaluate differences in phytochemical profiles between in vitro-derived and wild plants. By performing comparative GC–MS identification and GC–FID relative quantification, this study aimed to characterize differences in phytochemical profiles among the tested PGR treatments and between in vitro-derived and wild plant material. Particular emphasis was placed on the relative proportion of nerolidol, with the findings intended to inform future investigations of the biotechnological potential and conservation of this endemic species.

## 2. Results

### 2.1. Shoot Induction, Multiplication and Rooting Responses

In the initial cytokinin screening phase, explants cultured on PGR-free control medium (0) and kinetin-supplemented medium showed negligible development. In contrast, 6-benzylaminopurine (BAP) demonstrated a superior morphogenetic response. The highest shoot induction and elongation were recorded on MS medium containing BAP (1.0 mg/L), which proved significantly more effective than all other tested concentrations (Table 1). Statistical analysis confirmed significant differences across treatments for both shoot number (*F* = 37.241, *df*_1_ = 6, *df*_2_ = 203, *p* < 0.0001) and shoot length (*F* = 27.375, *df*_1_ = 6, *df*_2_ = 203, *p* < 0.0001).

Consistent with the sequential optimization strategy, treatments in the main experimental matrix that failed to induce shoot proliferation (e.g., MS + 4.0 mg/L NAA or IAA) were not continued beyond the initial 30-day culture period; however, their recorded non-responsive values were retained in the dataset and included in the statistical comparisons presented in Table 2. Conversely, responsive cultures were maintained through three consecutive 30-day subcultures (90 days in total) to obtain established and actively growing cultures, with quantitative data recorded at the end of the third subculture (Day 90) to represent their regenerative performance.

**Figure 2:**
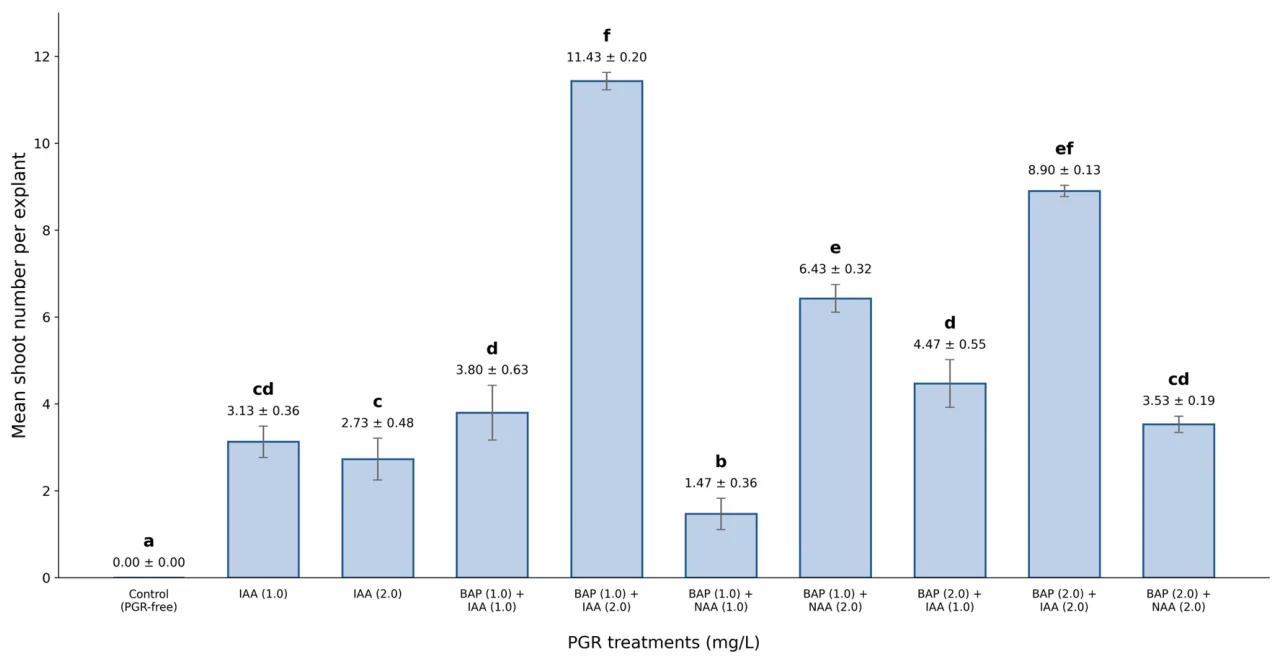
Shoot proliferation of *Stachys pinardii* cultured on different PGR combinations after the third subculture. Vertical bars represent standard error (±SE). Different letters above the bars indicate significant differences according to Duncan’s Multiple Range Test at α = 0.05.

Table 1 presents the cytokinin screening results, whereas Table 2 presents the responses to auxin treatments and BAP–auxin combinations evaluated for shoot multiplication. In the 27-group dataset used for the analysis of the main experimental matrix, significant differences were observed among treatments for shoot number (*F* = 136.319, *df*_1_ = 26, *df*_2_ = 783, *p* < 0.0001), shoot length (*F* = 95.093, *df*_1_ = 26, *df*_2_ = 783, *p* < 0.0001), and root number (*F* = 91.301, *df*_1_ = 26, *df*_2_ = 783, *p* < 0.0001). Table 2 presents the auxin-only and BAP–auxin treatment results from this dataset. Overall, the combinations containing the pre-optimized BAP concentration with IAA produced a stronger response than the corresponding BAP + NAA treatments. The highest mean shoot number was recorded on MS medium supplemented with BAP (1.0 mg/L) + IAA (2.0 mg/L) (Figure 2; Table 2).

A separate 2.0 mg/L BAP series was also evaluated to examine the effect of higher cytokinin levels on morphogenesis and subsequent phytochemical profiling. At 2.0 mg/L IAA, the treatment containing 2.0 mg/L BAP showed lower mean shoot number and shoot length than the corresponding treatment containing 1.0 mg/L BAP (Table 2). 

The relative ranking of the most responsive treatments remained similar across the three subculture passages, with BAP + IAA combinations showing higher morphogenetic responses than the corresponding BAP + NAA treatments. The multiplication rate increased progressively across the three subcultures, with the highest values recorded at the end of the third subculture (Day 90). In contrast, high auxin concentrations (4.0 mg/L IAA or NAA) caused severe growth inhibition and vitrification, with tissue deterioration becoming evident within 8.2 ± 0.5 days (mean ± SE).

Some Phase 2 combinations also showed direct rooting responses. In particular, BAP (1.0 mg/L) + IAA (2.0 mg/L) and BAP (1.0 mg/L) + NAA (2.0 mg/L) both achieved 100.00% rooting frequency in the multiplication medium (Table 2), indicating that the optimized PGR combination also supported early rhizogenic activity. For the dedicated rooting stage, healthy microshoots harvested from established and actively growing multiplication cultures after the third subculture were transferred to IBA-containing medium. Rooting responses differed significantly among treatments (*F* = 110.946, *df*_1_ = 2, *df*_2_ = 87, *p* < 0.0001). The highest rooting frequency (100.00%) and root number (3.63 ± 0.09) were obtained on MS medium supplemented with IBA (1.0 mg/L), whereas IBA (0.5 mg/L) resulted in only 33.33% rooting and IBA (2.0 mg/L) completely inhibited root formation (Table 3). 

**Figure 3:**
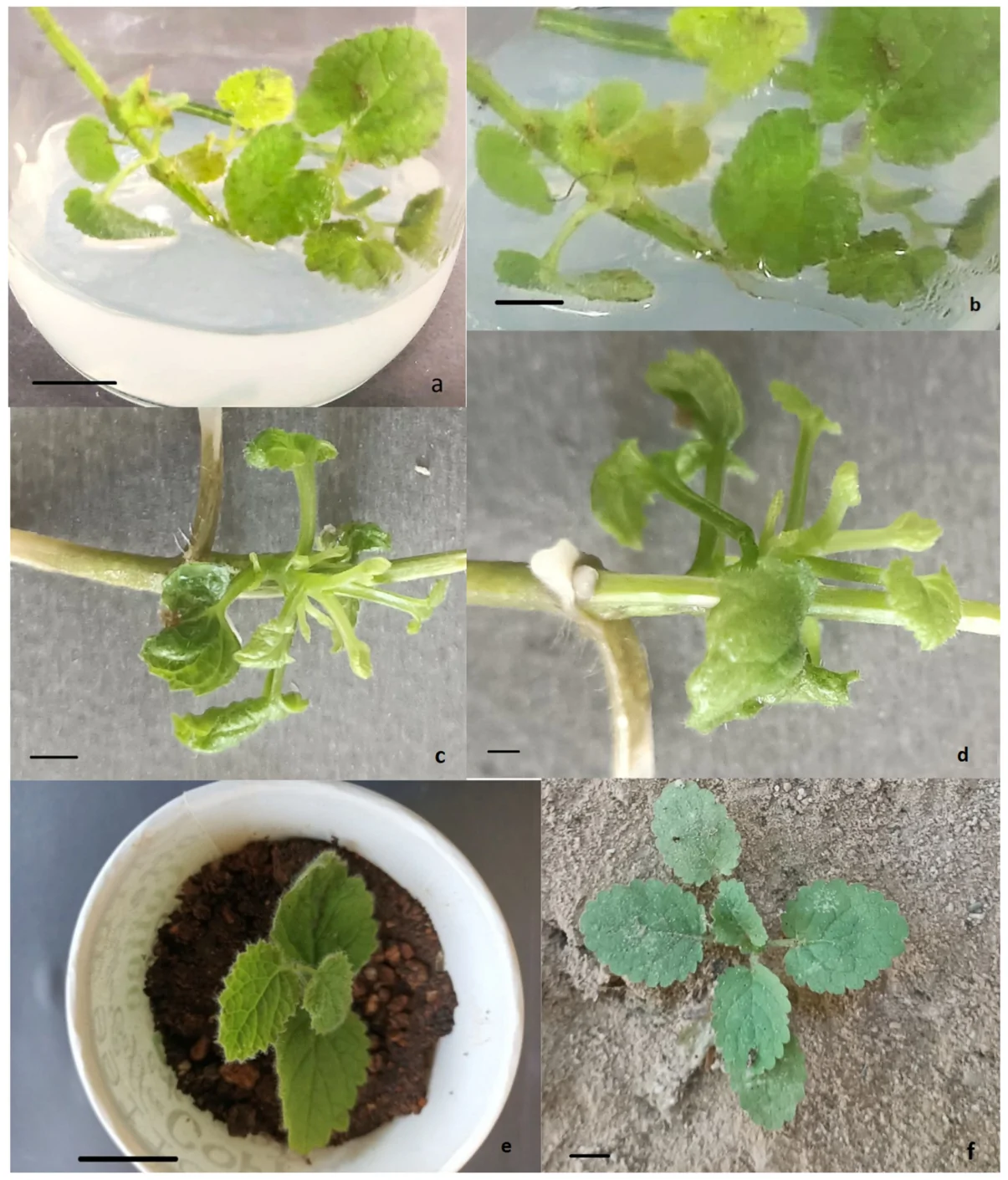
Representative stages of in vitro propagation of *Stachys pinardii:* (**a**) representative view of proliferation of nodal explants cultured on MS medium supplemented with BAP (1.0 mg/L) and IAA (2.0 mg/L) after the third subculture passage; (**b**) representative view of rooting in a regenerated shoot following transfer to IBA-supplemented rooting medium; (**c**,**d**) representative views of a regenerated explant showing multiple shoot formation from opposite sides; (**e**) acclimatization and hardening of in vitro-derived plantlets following transfer to small pots; (**f**) establishment of acclimatized plants under natural habitat conditions. Scale bars = 1.0 cm.

These results indicate that 1.0 mg/L IBA was the most effective concentration tested for rooting of *S. pinardii* microshoots. A total of 38 plantlets with well-developed roots and green shoots were transferred to acclimatization conditions. Of these, 32 survived, corresponding to a survival rate of 84.21% (Figure 3).

### 2.2. GC–MS and GC–FID Analysis of Hexane Extracts

GC–MS identification and GC–FID relative quantification of hexane extracts revealed differences in phytochemical profiles between in vitro-derived and wild-grown plants (Table 4). The major compound identified was nerolidol, which was detected at 45.4% in plants cultured on MS medium containing BAP (1.0 mg/L) + IAA (2.0 mg/L), compared to 10.3% in wild-grown plants. In the BAP (2.0 mg/L) + IAA (2.0 mg/L) treatment, the relative proportion of nerolidol was 2.1%.

Carvacrol (12.0%) and phytol (6.8%) were detected in wild plants but were not detected in plants cultured with BAP (1.0 mg/L) + IAA (2.0 mg/L). Palmitic acid (9.1–9.5%) and α-linolenic acid derivatives (15.4–16.1%) were detected in the BAP (2.0 mg/L) + IAA (2.0 mg/L) treatment and wild plants but were not detected in the optimized BAP (1.0 mg/L) + IAA (2.0 mg/L) treatment.

### 2.3. GC–MS and GC–FID Analysis of Methanol Extracts

Methanol extracts showed a dominance of polar metabolites including organic acids, sugars, and fatty acid derivatives (Table 5). The highest relative abundances of benzoic acid (10.4%) and glycerol (14.0%) were observed in plants cultured on MS medium supplemented with BAP (2.0 mg/L) + IAA (2.0 mg/L).

Sugars such as β-D-glucopyranose (14.8%) and α-D-glucopyranose (10.7%) were also prominent in in vitro samples. Fatty acids including palmitic acid (18.6%) and α-linolenic acid derivatives (12.9%) were among the major constituents. The detected sugars may reflect endogenous plant carbohydrates; in the in vitro-derived samples, their relative profiles may also have been influenced by the sucrose-containing culture medium. Because the present analysis cannot distinguish between these potential sources, the origin of individual sugars cannot be assigned unequivocally. The unidentified fraction in the methanol extracts reflects chromatographic peaks that could not be reliably assigned using the available commercial GC–MS libraries under the applied identification criteria. Differences in metabolite distribution were observed between wild and cultured plants across both polar and non-polar fractions.

The morphogenetic responses corresponding to the in vitro samples (Hexane-1, Hexane-2, Methanol-1, and Methanol-2) are presented in Table 2, providing a direct link between the regeneration performance of each PGR treatment and its corresponding phytochemical profile.

## 3. Discussion

This study provides the first comprehensive report on the micropropagation and comparative phytochemical profiling of *Stachys pinardii*, an endemic species of Turkey. The findings indicate that PGR treatments are associated with differences in morphogenetic responses, consistent with previous tissue culture studies in Lamiaceae species [13,14,15]. Differences in phytochemical profiles were also observed among the PGR treatments evaluated in the present study. One of the key findings of this study is the association between morphogenetic development and differences in phytochemical profiles in *S. pinardii*. The marked shift from a profile containing carvacrol in wild-grown plants to a nerolidol-enriched profile (45.4%) in vitro indicates that the optimized culture conditions were associated with substantial differences in terpenoid composition. These findings further indicate that the combined application of BAP and IAA was associated not only with enhanced organogenic responses but also with a different terpenoid profile.

In the present study, cytokinin supplementation, particularly 6-benzylaminopurine (BAP), significantly promoted shoot induction, while its combination with auxins enhanced regeneration efficiency. The highest regeneration response was obtained with BAP (1.0 mg/L) + IAA (2.0 mg/L), suggesting a beneficial interaction between cytokinins and auxins during shoot proliferation. Similar cytokinin-dependent morphogenetic responses have been reported in *Ocimum gratissimum*, *O. kilimandscharicum*, and *Stachys cretica* subsp. *kutahyensis* [14,15,17]. At 2.0 mg/L IAA, the treatment containing 1.0 mg/L BAP produced higher mean shoot number and shoot length than the treatment containing 2.0 mg/L BAP (Figure 2; Table 2). The combination of BAP (1.0 mg/L) and IAA (2.0 mg/L) was identified as the optimal treatment for shoot proliferation. This reduction in morphogenetic response at higher cytokinin levels suggests an inhibitory effect of supra-optimal plant growth regulator (PGR) concentrations, a phenomenon well-documented in other Lamiaceae taxa. For instance, in both *Ocimum gratissimum* and *Ocimum kilimandscharicum*, the highest shoot proliferation was reported at 1.0 mg/L BAP, whereas higher BAP concentrations resulted in lower shoot proliferation [14,15]. Although such concentration-dependent inhibitory responses to BAP have been reported in other Lamiaceae species, the present study did not investigate endogenous hormone levels or the physiological mechanisms underlying this response; therefore, the observed reduction in shoot proliferation at higher BAP concentrations should not be attributed to a specific hormonal mechanism. Under the conditions tested, *S. pinardii* showed a maximum of 11.43 ± 0.20 shoots per explant with a shoot length of 3.36 ± 0.05 cm on MS medium containing BAP (1.0 mg/L) + IAA (2.0 mg/L). For comparison, Akın et al. [17] reported a maximum of 4.60 shoots per explant and a shoot length of 2.70 cm for *S. cretica* subsp. *kutahyensis* on MS medium containing 2.0 mg/L BAP. Although direct comparison between species should be made cautiously because of differences in explant source and culture conditions, these data provide a quantitative context for the regeneration response observed in *S. pinardii*.

Root induction was effectively achieved using indole-3-butyric acid (IBA), consistent with its well-established role in adventitious root formation in medicinal plants. The superior rooting response observed in this study is consistent with previous reports of auxin-mediated rooting in Lamiaceae taxa [12,16]. In contrast, higher auxin concentrations inhibited root development, suggesting a concentration-dependent effect of PGRs on organogenesis.

A key finding of this study is the marked difference in phytochemical profiles between in vitro-cultured and wild plants. GC–MS identification combined with GC–FID relative quantification revealed substantial differences in metabolite profiles, particularly in the terpenoid fraction. The most prominent difference was the higher relative proportion of nerolidol in the optimized in vitro treatment (45.4%) compared with wild-grown plants (10.3%). This difference suggests that in vitro culture conditions, together with specific PGR treatments, may be associated with changes in terpenoid profiles. Plant isoprenoid metabolism comprises highly regulated and interconnected pathways that contribute to the diversity of terpenoid compounds [25]. Because the in vitro cultures originated from nodal explants collected from the same donor plants used for the wild samples, this sampling design reduced the potential influence of differences in plant origin when evaluating phytochemical differences associated with in vitro culture conditions and PGR treatments. The use of a two-phase sequential optimization strategy together with the experimental design allowed the higher relative proportion of nerolidol (45.4%) to be associated with the BAP (1.0 mg/L) + IAA (2.0 mg/L) treatment while minimizing uncertainty regarding the origin of the analyzed plant material. However, the reason for the lower relative proportion of nerolidol in wild-grown plants (10.3%) compared with the optimized in vitro treatment (45.4%) cannot be determined from the present data. The observed difference may reflect the combined influence of culture environment, PGR treatment, developmental status, and other physiological or environmental factors. Therefore, the present results demonstrate a nerolidol-enriched relative phytochemical profile under the optimized in vitro conditions but do not establish that in vitro culture increased the absolute production or yield of nerolidol.

In wild-grown plants, the major constituents of the hexane extract included α-linolenic acid derivatives (15.4%), hentriacontane (14.6%), carvacrol (12.0%), linoleic acid derivatives (11.6%), nerolidol (10.3%), palmitic acid (9.1%), geranyl-*p*-cymene (9.0%), and phytol (6.8%). Among these constituents, carvacrol is particularly noteworthy because of its well-documented biological and pharmacological properties [26]. The relative distribution of these constituents also differed among the two in vitro treatments and the wild-grown plants. Carvacrol, which represented 12.0% of the wild-grown profile, was detected at a lower relative percentage (3.1%) in the high-cytokinin treatment [BAP (2.0 mg/L) + IAA (2.0 mg/L)] and was not detected in the optimized treatment [BAP (1.0 mg/L) + IAA (2.0 mg/L)]. Similarly, palmitic acid, linoleic acid, and α-linolenic acid derivatives occurred at relatively high proportions in the wild-grown and high-cytokinin profiles but were not detected in the optimized treatment. In contrast, the optimized treatment was characterized by a nerolidol-enriched relative profile. These differences indicate a redistribution of relative phytochemical composition among the tested groups rather than an overall increase or decrease in metabolite production. Environmental conditions are known to influence specialized metabolite profiles; however, the present study was not designed to determine whether specific environmental factors were responsible for the observed differences between wild-grown and in vitro-derived plants [27]. Therefore, these differences should be interpreted primarily as changes in relative phytochemical profiles rather than as direct evidence of altered biosynthetic activity or changes in the underlying biosynthetic mechanisms.

Furthermore, higher BAP concentrations were associated with increased proportions of primary metabolites, including fatty acids and sugars, suggesting a shift in the relative distribution of primary and specialized metabolites. Similar PGR-dependent metabolic differences have been reported in *Melissa officinalis*, where PGR treatments affected total phenol content and metabolic profiles [28]. Similar variation in specialized metabolite composition has also been documented within the Lamiaceae, particularly in *Stachys* species, in which substantial differences in volatile and other phytochemical constituents have been reported among taxa and extracts [4,5,6,29,30]. Together with the PGR-dependent metabolic differences reported for in vitro-cultured *Melissa officinalis* [28], these observations provide a more specific comparative context for the differences in phytochemical profiles observed in *S. pinardii* under the tested culture conditions.

The choice of extraction solvent was based on the polarity of the target compounds, thereby influencing the recovered metabolite profile. The hexane fractions contained constituents such as nerolidol and geranyl derivatives, whereas the subsequent methanol fractions contained polar constituents, including organic acids and saccharides. Because methanol extraction followed hexane extraction of the same plant material, these profiles reflect both solvent polarity and extraction sequence, rather than a direct comparison of solvent extraction efficiency. This chemical differentiation is consistent with studies in related *Stachys* taxa showing that the recovered phytochemical profile may vary according to the extraction solvent [29,30].

From a biotechnological perspective, the established micropropagation system provides an efficient tool for both ex situ conservation and the production of uniform plant material of *S. pinardii*. Micropropagation may contribute to reducing harvesting pressure on natural populations while enabling the controlled production of plant material with different phytochemical profiles, which is particularly important for the sustainable utilization of medicinal plants [31,32]. Interactions among monoterpenoid compounds, including synergistic effects, have been reported in biological activity assays [33]. Therefore, the established protocol may provide a basis for future investigations into the potential biotechnological applications of this plant material.

Among the metabolites identified, nerolidol emerged as the most promising compound from a biotechnological perspective because of its broad industrial relevance in pharmaceutical, cosmetic, fragrance, and food applications. Its antimicrobial, antioxidant, anti-inflammatory, and insect-repellent activities have been widely documented [21]. The approximately fourfold higher relative proportion of nerolidol observed under the optimized culture conditions (45.4% versus 10.3% in wild plants) indicates that the optimized PGR combination was associated with a nerolidol-enriched phytochemical profile. Although the present study did not evaluate the economic feasibility of large-scale production, the simultaneous achievement of efficient micropropagation and a nerolidol-enriched phytochemical profile suggests that the established protocol may provide a basis for future investigations of PGR-associated changes in phytochemical profiles [28]. Importantly, the in vitro cultures in the present study were not established to quantify biomass productivity or absolute specialized metabolite yield; rather, they were used to generate clonally propagated plant material under defined PGR conditions for comparative phytochemical profiling with the corresponding wild material. Nevertheless, further studies addressing biomass productivity, scale-up using bioreactor systems, metabolite yield optimization, and techno-economic feasibility will be required before commercial application can be considered. 

It should also be noted that the GC–FID data represent relative peak-area proportions rather than absolute metabolite concentrations; therefore, the observed differences should be interpreted as differences in relative phytochemical composition. However, although metabolite profiling clearly demonstrates differences between in vitro-derived and wild plant material, the present findings remain descriptive at the metabolite level. Each group was represented by pooled composite plant material, and the three analyses were repeated measurements of the same extract rather than independent biological replicates. Consequently, the data do not quantify biological variability within groups, and the observed profile differences cannot be generalized statistically to the corresponding plant populations. Further molecular investigations, including gene expression and pathway-level analyses, are required to elucidate the regulatory mechanisms underlying these differences in phytochemical profiles. Such approaches would be required to determine whether the observed differences in phytochemical profiles are associated with specific changes in biosynthetic pathways.

## 4. Materials and Methods

### 4.1. Plant Material and Sample Collection

The upper shoots of *Stachys pinardii* were collected from its natural habitat on limestone cliffs in Geyikbayırı, Antalya, Turkey (C3 square) during the full flowering stage in July 2022. The species was identified by Prof. Muhittin Dinç, Hatice Üstüner, and Vedat Kadir Özkan based on the taxonomic descriptions and identification keys provided in the relevant literature [3,7].

Voucher specimens were deposited in the Selçuk University Faculty of Science Herbarium (Konya, Turkey) (Voucher No: M. Dinç, H. Üstüner & V.K. Özkan, 3662). For phytochemical analysis, wild-grown vegetative upper shoot tissues, excluding flowers, were collected from ten individual wild donor plants to incorporate donor-level variation within the sampled population while providing corresponding source plants for the establishment of ten independent clonal in vitro lines. The ten donor plants were not intended to constitute a statistically representative sample of the entire natural population, but rather to provide a defined set of biological sources for the paired wild and in vitro-derived comparative material. The plant material was then air-dried in a shaded, low-humidity environment prior to extraction. Various reproductive mechanisms promoting outcrossing occur in Lamiaceae and can contribute to genetic variation among seed-derived individuals [34]. To minimize genotype-related variation between the compared sample groups and to ensure that each in vitro culture originated from its corresponding wild donor plant, clonal propagation via nodal segments was used instead of seed germination. Accordingly, ten independent clonal in vitro lines, each derived from a different donor plant, were maintained throughout the micropropagation experiments. For biochemical standardization, equal weights of vegetative upper shoot tissues, excluding flowers, obtained from the same ten donor plants and their corresponding clonally propagated in vitro lines were pooled separately into composite samples before extraction for the wild and in vitro groups. This approach was used to minimize the influence of donor plant-related genetic variation on the comparative material, thereby facilitating the evaluation of differences in phytochemical profiles associated with in vitro culture conditions and the applied PGR treatments.

### 4.2. Surface Sterilization and In Vitro Culture Establishment

Nodal explants were washed under running tap water for 30 min, followed by treatment with a few drops of Tween 20 to remove surface impurities. Surface sterilization was performed in a laminar flow cabinet by first immersing the explants in 70% (*v*/*v*) ethanol for 1 min, followed by 20% (*v*/*v*) sodium hypochlorite (containing 4.6% active chlorine) for 15 min, consistent with surface-sterilization approaches used for in vitro culture of Lamiaceae taxa [16,35]. Subsequently, the explants were rinsed three times with sterile distilled water to remove residual chlorine. No systemic fungicides or bacteriostatics were used as the optimized protocol yielded a high decontamination success rate (96.7%). Following culture establishment, 93.3% of the explants survived, and the surviving explants exhibited uniform growth under in vitro conditions. The sterilized explants were inoculated onto Murashige and Skoog (MS) basal medium [36] supplemented with sucrose (30 g/L) and plant agar (7 g/L; Duchefa Biochemie, Haarlem, The Netherlands). The pH of all media was adjusted to 5.7–5.8 using 1 N NaOH or HCl prior to autoclaving at 121 °C (1.05 kg/cm^2^) for 20 min.

### 4.3. Shoot Proliferation and Rooting Conditions

The micropropagation experiments included cytokinin screening (Table 1) and evaluation of auxin treatments and BAP–auxin combinations for shoot multiplication (Table 2). Phase 1 (Preliminary screening): Nodal explants were cultured on MS medium supplemented with various BAP and KIN concentrations to evaluate cytokinin-dependent responses. Responsive cultures were maintained through three consecutive 30-day subcultures (90 days total), and their responses were recorded at the end of the third subculture (Table 1). Phase 2 (Main experimental matrix): The statistical analysis of the main experimental matrix included 27 groups, with 30 observations per group (810 observations in total). Table 2 presents the auxin-only and BAP–auxin treatment results; the PGR-free and BAP-only groups included in the analysis are not displayed in this table. The combined effects of the previously identified optimal BAP concentration (1.0 mg/L) and auxins (IAA, NAA) were investigated over another three 30-day subcultures (90 days total) (Table 2). This phase included an additional 2.0 mg/L BAP treatment series to evaluate the effects of a higher BAP concentration on morphogenetic responses and specialized metabolite profiles. Traceability and Labeling: Throughout Phase 2, all culture vessels were meticulously labeled according to their specific PGR concentrations. The identity of the cultures was maintained according to the source jar labels, allowing plant material used for phytochemical analyses to remain traceable to the corresponding PGR treatments. Healthy microshoots selected for the rooting stage also retained their source-treatment identity. Rooting and Acclimatization: Rooting was performed as a single 30-day stage on IBA-supplemented medium (Table 3). The total duration of the propagation process from the initiation of Phase 2 to the completion of acclimatization was 180 days: 90 days for shoot multiplication, 30 days for rooting, and 60 days for acclimatization. The optimized micropropagation protocol consisted of three developmental stages: culture establishment (initiation), shoot multiplication, and rooting. The culture establishment (initiation) stage corresponded to the first 30-day subculture, after which shoot multiplication continued through two additional 30-day subcultures before the rooting stage. Proliferation experiments were carried out in 100 mL glass culture vessels containing 30 mL of medium, with three nodal explants per vessel and a total of ten vessels per treatment (*n* = 30). Cultures were maintained in a growth chamber at 25 ± 2 °C under a 16 h light/8 h dark photoperiod with cool-white fluorescent light (PPFD: 40–50 μmol m^−2^ s^−1^). Regenerated shoots that remained green, turgid, and actively growing were considered viable. For rooting, microshoots (≥2 cm) were transferred to MS medium supplemented with various IBA concentrations (0.5–2.0 mg/L). The IBA concentrations (0.5, 1.0, and 2.0 mg/L) were selected to evaluate the rooting response across a low-to-high concentration range and to identify an effective concentration for root induction in *S. pinardii*. Rooting observed in some BAP + auxin treatments during the multiplication phase occurred concurrently with shoot proliferation, whereas the separate IBA treatment was used to evaluate rooting of regenerated microshoots in a dedicated rooting stage.

### 4.4. Acclimatization 

The acclimatization procedure was adapted from approaches reported for other Lamiaceae taxa [12,15]. For the acclimatization phase, fully rooted plantlets were carefully extracted from the culture vessels and their roots were gently washed to remove residual agar using lukewarm tap water. Subsequently, the plantlets were transplanted into small pots containing a sterilized substrate of peat moss and perlite (1:1, *v*/*v*). No chemical fertilizers were incorporated into the peat–perlite (1:1, *v*/*v*) substrate during the initial acclimatization period. To facilitate the transition to autotrophic growth without causing nutrient-related osmotic stress, the plantlets received initial irrigation with a sucrose-free, 1/8-strength MS basal salt solution adjusted to pH 5.8. High relative humidity was maintained by enclosing the pots in transparent polyethylene bags during the primary hardening stage. The plantlets were maintained in a controlled growth environment at 25 ± 2 °C with a 16 h light/8 h dark photoperiod for 14 days before being transferred to greenhouse conditions. A total of 38 rooted plantlets were transferred for acclimatization. Survival was calculated as the number of surviving plantlets divided by the total number transferred, multiplied by 100.

### 4.5. Phytochemical Extraction, GC–MS, and GC–FID Analysis

To characterize the specialized metabolites, air-dried vegetative upper shoot tissues, excluding flowers, were used for phytochemical analysis. The same plant-part criterion was applied to both the in vitro-derived and wild-grown samples to ensure consistency in the comparative phytochemical profiling. Comparative phytochemical profiling was performed using two in vitro treatment groups: MS medium supplemented with BAP (1.0 mg/L) + IAA (2.0 mg/L) (optimized proliferation group) and MS medium supplemented with BAP (2.0 mg/L) + IAA (2.0 mg/L) (high-cytokinin comparison group). For the wild-grown group, a total of 55 g of air-dried aerial material (dry weight) was collected and processed for phytochemical analysis using the same sampling and extraction strategy; this amount represents the analytical material collected rather than a measurement of biomass productivity. For each in vitro treatment group, a total bulk of 15 g dry weight was obtained from surplus microshoots that were not required for the rooting and acclimatization experiments. This pooled biomass was pulverized using a Retsch SM 100 mill. Each extract obtained from the pooled plant material was analyzed in triplicate. The plant material was first extracted with hexane, after which the remaining plant residue was extracted with methanol. Hexane and methanol were selected as complementary extraction solvents because of their different polarities, allowing the recovery of a broader range of non-polar and polar constituents, respectively, for comparative phytochemical profiling. This strategy ensured that sufficient high-quality material was available for phytochemical profiling while maintaining the required number of plantlets for rooting and acclimatization studies. The plant material used for these analyses originated from the composite samples prepared from the same donor plants and their corresponding clonally propagated in vitro lines, as described in the Plant Material and Sample Collection section. 

Sufficient solvent was added to fully cover the plant material, which was then macerated for 24 h with intermittent shaking and filtered through filter paper using a funnel. For each solvent, the maceration and filtration procedure was performed three times, using fresh solvent for each successive cycle, with each maceration lasting 24 h. Extracts were evaporated to dryness under reduced pressure using a rotary evaporator at 45 °C and derivatized with a 1:1 mixture of N-methyl-N-(trimethylsilyl) trifluoroacetamide (MSTFA) and chloroform at 65 °C for 60 min prior to GC–MS analysis. Derivatization was performed to convert polar compounds containing active hydrogen functional groups into their more volatile trimethylsilyl derivatives, thereby improving their suitability for GC analysis. Chloroform was not used as an extraction solvent; it was used only as a co-solvent with MSTFA during the derivatization step prior to GC analysis. GC–MS analysis was performed using an Agilent 5975C Inert XL EI/CI MSD system operating in electron ionization (EI) mode and equipped with an HP-5MS capillary column (30 m × 0.25 mm i.d., 0.25 μm film thickness; 5% phenyl, 95% methylpolysiloxane). The oven temperature was programmed as follows: 60 °C for 1 min, increased to 246 °C at 3 °C min^−1^, and held isothermally for 30 min. Helium was used as the carrier gas at a constant flow rate of 0.9 mL min^−1^. Samples (1 μL) were injected in split mode (1:50). The ion source temperature was maintained at 230 °C, while the injector and transfer line temperatures were set at 250 °C. Electron ionization was performed at 70 eV, and mass spectra were acquired over the range of *m/z* 35–450. 

Compound identification was based on a dual-criteria approach: (i) comparison of electron ionization (EI) mass spectra with the Wiley 8th Edition and NIST17 mass spectral libraries, and (ii) calculation of Relative Retention Indices (RRIs) using a homologous series of *n*-alkanes (C_8_–C_40_), followed by comparison with published literature values [37,38]. Relative quantification of the identified compounds was performed by gas chromatography with flame ionization detection (GC–FID) using an Agilent 6890N GC system. The FID detector temperature was maintained at 300 °C. To ensure identical chromatographic elution between qualitative (GC–MS) and quantitative (GC–FID) analyses, simultaneous automatic injections were carried out under identical operating conditions using two identical HP-5MS capillary columns. Relative percentages of the identified compounds were calculated by electronic integration of the peaks in the FID chromatograms, following the procedure of Servi et al. [37]. The reported values are presented as mean relative percentages (%) ± SD calculated from three repeated GC–FID analyses of the same extract (*n* = 3). These are analytical repeats; the SD reflects analytical repeatability rather than variation among independent extractions or biological samples. Because GC–FID quantification was based on peak-area normalization without an internal standard or external calibration standards, calibration curves were not constructed and method detection or quantification limits (MDL/LOD/LOQ) were not determined. Accordingly, “not detected” (nd) indicates that no corresponding peak was detected under the applied analytical conditions; a formal detection limit was not determined. Therefore, the reported percentages represent relative proportions within the detected compound profile rather than absolute concentrations.

### 4.6. Statistical Analysis

All experiments were conducted using a completely randomized design. Morphogenetic data are presented as mean ± standard error (SE) (*n* = 30), whereas GC–FID data are presented as mean relative peak-area percentages (%) ± standard deviation (SD) based on three repeated analyses of the same extract (*n* = 3). The normality of the morphogenetic data was evaluated using the Shapiro–Wilk test. Treatment differences in morphogenetic parameters were evaluated by one-way analysis of variance (ANOVA), followed by Duncan’s Multiple Range Test [39] and Tukey’s Honestly Significant Difference (HSD) post-hoc tests at α = 0.05. GC–FID relative peak-area percentages were summarized descriptively as mean ± SD; no inferential comparisons among phytochemical sample groups were performed. Both post-hoc procedures were applied to assess the consistency of treatment-group separation across two multiple-comparison approaches, with Tukey’s HSD providing a more conservative control of family-wise error and Duncan’s test providing the multiple-range grouping commonly used for treatment comparisons in plant tissue culture studies. All statistical analyses were performed using IBM SPSS Statistics version 23.0.

## 5. Conclusions

This study establishes an efficient three-stage micropropagation protocol for the endemic species *Stachys pinardii*. The combination of BAP (1.0 mg/L) and IAA (2.0 mg/L) produced the highest shoot proliferation (11.43 ± 0.20 shoots per explant). During acclimatization, 32 of the 38 transferred plantlets survived (84.21%). Beyond establishing an efficient propagation protocol, the findings indicate that in vitro culture systems can provide a controlled platform for investigating differences in specialized metabolite profiles. This integrated approach highlights the potential value of the established protocol for both biotechnological investigation and ex situ conservation of this endemic species. Beyond propagation efficiency, the study revealed differences in phytochemical composition, particularly a higher relative proportion of nerolidol in the optimized in vitro treatment (45.4%) compared with wild-grown plants (10.3%). 

As the present phytochemical comparison is descriptive and did not assess biomass productivity, absolute metabolite yields, scale-up, or economic feasibility, further studies addressing these aspects, together with molecular-level analyses, are required before the broader biotechnological applicability of the established protocol can be evaluated.

Overall, this study provides a practical micropropagation protocol for *S. pinardii* and a comparative characterization of phytochemical profiles in in vitro-derived and wild-grown plant material, providing a basis for further conservation-oriented and biotechnological investigations.

## Figures and Tables

**Table 1:** Effects of different cytokinin treatments on shoot induction and multiplication from nodal explants, recorded at the end of the third subculture for responsive cultures.

Plant Growth Regulators (mg/L)	Shoot Number (per Explant)	Shoot Length (cm)	Root Number (per Explant)
(0)	_a_ 0.00 ± 0.00 ^a^	_a_ 0.00 ± 0.00 ^a^	0.00 ± 0.00
BAP (0.5)	_a_ 0.00 ± 0.00 ^a^	_a_ 0.00 ± 0.00 ^a^	0.00 ± 0.00
BAP (1.0)	_b_ 2.40 ± 0.34 ^b^	_c_ 1.19 ± 0.17 ^b^	0.00 ± 0.00
BAP (2.0)	_a_ 0.23 ± 0.11 ^a^	_ab_ 0.20 ± 0.09 ^a^	0.00 ± 0.00
KIN (0.5)	_a_ 0.33 ± 0.12 ^a^	_b_ 0.31 ± 0.11 ^a^	0.00 ± 0.00
KIN (1.0)	_a_ 0.00 ± 0.00 ^a^	_a_ 0.00 ± 0.00 ^a^	0.00 ± 0.00
KIN (2.0)	_a_ 0.00 ± 0.00 ^a^	_a_ 0.00 ± 0.00 ^a^	0.00 ± 0.00
ANOVA	*F* = 37.241 *	*F* = 27.375 *	*F* = -

* Values are expressed as mean ± SE (*n* = 30). Letters in the bottom left represent the Duncan test, and letters in the top right represent the Tukey HSD test. Different letters indicate significant differences according to the corresponding post-hoc test at α = 0.05. Overall ANOVA results: *p* < 0.0001, *df*_1_ = 6, *df*_2_ = 203.

**Table 2:** Effects of IAA and NAA applied alone or in combination with BAP (1.0 or 2.0 mg/L) on shoot regeneration and morphogenetic responses of *Stachys pinardii*, recorded at the end of the third subculture for responsive cultures.

Plant Growth Regulators (mg/L)	Shoot Number (per Explant)	Shoot Length (cm)	Root Number (per Explant)	Rooting Percentage (%)
NAA (0.5)	_a_ 0.00 ± 0.00 ^a^	_a_ 0.00 ± 0.00 ^a^	_a_ 0.00 ± 0.00 ^a^	0.00
NAA (1.0)	_a_ 0.27 ± 0.15 ^ab^	_a_ 0.15 ± 0.08 ^a^	_a_ 0.00 ± 0.00 ^a^	0.00
NAA (2.0)	_a_ 0.57 ± 0.28 ^ab^	_a_ 0.20 ± 0.09 ^a^	_a_ 0.00 ± 0.00 ^a^	0.00
NAA (4.0)	_a_0.00 ± 0.00 ^a^	_a_ 0.00 ± 0.00 ^a^	_a_ 0.00 ± 0.00 ^a^	0.00
IAA (0.5)	_a_ 0.00 ± 0.00 ^a^	_a_ 0.00 ± 0.00 ^a^	_a_ 0.00 ± 0.00 ^a^	0.00
IAA (1.0)	_cd_ 3.13 ± 0.36 ^d^	_b_ 1.14 ± 0.12 ^bc^	_b_ 0.33 ± 0.14 ^ab^	16.67
IAA (2.0)	_c_ 2.73 ± 0.48 ^cd^	_b_ 0.88 ± 0.15 ^b^	_b_ 0.50 ± 0.16 ^b^	26.67
IAA (4.0)	_a_ 0.00 ± 0.00 ^a^	_a_ 0.00 ± 0.00 ^a^	_a_ 0.03 ± 0.03 ^a^	3.33
BAP (1.0) + IAA (0.5)	_a_ 0.00 ± 0.00 ^a^	_a_ 0.00 ± 0.00 ^a^	_a_ 0.00 ± 0.00 ^a^	0.00
BAP (1.0) + IAA (1.0)	_d_ 3.80 ± 0.63 ^d^	_c_ 1.48 ± 0.24 ^c^	_b_ 0.50 ± 0.14 ^b^	33.33
BAP (1.0) + IAA (2.0)	_f_ 11.43 ± 0.20 ^f^	_e_ 3.36 ± 0.05 ^e^	_c_ 2.00 ± 0.00 ^c^	100.00
BAP (1.0) + IAA (4.0)	_a_ 0.07 ± 0.07 ^a^	_a_ 0.00 ± 0.00 ^a^	_a_ 0.10 ± 0.07 ^a^	6.67
BAP (1.0) + NAA (0.5)	_a_ 0.00 ± 0.00 ^a^	_a_ 0.00 ± 0.00 ^a^	_a_ 0.00 ± 0.00 ^a^	0.00
BAP (1.0) + NAA (1.0)	_b_ 1.47 ± 0.36 ^bc^	_b_ 0.96 ± 0.24 ^b^	_b_ 0.50 ± 0.14 ^b^	33.33
BAP (1.0) + NAA (2.0)	_e_ 6.43 ± 0.32 ^e^	_d_ 2.77 ± 0.04 ^d^	_c_ 2.00 ± 0.00 ^c^	100.00
BAP (1.0) + NAA (4.0)	_a_ 0.00 ± 0.00 ^a^	_a_ 0.00 ± 0.00 ^a^	_a_ 0.00 ± 0.00 ^a^	0.00
BAP (2.0) + IAA (0.5)	_a_ 0.00 ± 0.00 ^a^	_a_ 0.00 ± 0.00 ^a^	_a_ 0.00 ± 0.00 ^a^	0.00
BAP (2.0) + IAA (1.0)	_d_ 4.47 ± 0.55 ^d^	_c_ 1.63 ± 0.20 ^c^	_b_ 0.50 ± 0.14 ^b^	33.33
BAP (2.0) + IAA (2.0)	_ef_ 8.90 ± 0.13 ^e^	_d_ 2.51 ± 0.01 ^d^	_c_ 2.00 ± 0.00 ^c^	100.00
BAP (2.0) + IAA (4.0)	_a_ 0.07 ± 0.07 ^a^	_a_ 0.00 ± 0.00 ^a^	_a_ 0.10 ± 0.07 ^a^	6.67
BAP (2.0) + NAA (0.5)	_a_ 0.00 ± 0.00 ^a^	_a_ 0.00 ± 0.00 ^a^	_a_ 0.00 ± 0.00 ^a^	0.00
BAP (2.0) + NAA (1.0)	_b_ 1.47 ± 0.36 ^bc^	_b_ 0.84 ± 0.21 ^b^	_a_ 0.13 ± 0.06 ^a^	13.33
BAP (2.0) + NAA (2.0)	_cd_ 3.53 ± 0.19 ^d^	_d_ 2.35 ± 0.03 ^d^	_c_ 1.37 ± 0.09 ^b^	100.00
BAP (2.0) + NAA (4.0)	_a_ 0.00 ± 0.00 ^a^	_a_ 0.00 ± 0.00 ^a^	_a_ 0.00 ± 0.00 ^a^	0.00
ANOVA	*F* = 136.319 *	*F* = 95.093 *	*F* = 91.301 *	not applicable

Values are expressed as mean ± SE (*n* = 30). Letters in the bottom left represent the Duncan test, and letters in the top right represent the Tukey HSD test. Different letters indicate significant differences according to the corresponding post-hoc test at α = 0.05. Overall ANOVA results: *p* < 0.0001, *df*_1_ = 26, *df*_2_ = 783. * Indicates *p* < 0.0001.

**Table 3:** Optimization of in vitro rooting: Effect of IBA concentrations on root development of *Stachys pinardii* microshoots after a 30-day rooting period.

Plant Growth Regulators (mg/L)	Root Number (per Explant)	Rooting Percentage (%)
IBA (0.5)	_b_ 1.10 ± 0.29 ^b^	33.33
IBA (1.0)	_c_ 3.63 ± 0.09 ^c^	100.00
IBA (2.0)	_a_ 0.00 ± 0.00 ^a^	0.00
ANOVA	*F* = 110.946 *	not applicable

Values are expressed as mean ± SE (*n* = 30). Letters in the bottom left represent the Duncan test, and letters in the top right represent the Tukey HSD test. Different letters indicate significant differences according to the corresponding post-hoc test at α = 0.05. Overall ANOVA results: *p* < 0.0001, *df*_1_ = 2, *df*_2_ = 87. * Indicates *p* < 0.0001.

**Table 4:** Compounds identified by GC–MS and relatively quantified by GC–FID in the hexane extract of *Stachys pinardii*. Hexane 1: plantlets cultured on MS medium supplemented with BAP (1.0 mg/L) and IAA (2.0 mg/L); Hexane 2: plantlets cultured on MS medium supplemented with BAP (2.0 mg/L) and IAA (2.0 mg/L); Hexane 3: plants collected from the wild. Values are expressed as mean relative percentages (%) ± SD from three repeated GC–FID analyses of the same extract (*n* = 3).

RT	RRI ^1^	RRI Lit. ^2^	Compounds	Hexane-1 (%)	Hexane-2 (%)	Hexane-3 (%)
13.809	1246	1244	Benzoic Acid, TMS derivative	nd	0.2 ± 0.03	nd
16.056	1328		Carvacrol, TMS derivative	nd	3.1 ± 0.03	12.0 ± 0.03
20.590	1506	1510	Hexanedioic acid, 2TMS derivative	nd	5.8 ± 0.04	nd
21.813	1559	1564	Nerolidol	45.4 ± 0.11	2.1 ± 0.03	10.3 ± 0.02
24.524	1676		Nerolidol, O-TMS	nd	2.7 ± 0.03	4.0 ± 0.02
30.062	1941	1962	Geranyl-α-terpinene	20.2 ± 0.07	nd	nd
27.065	1793	1807	Azelaic acid, 2TMS derivative	nd	0.3 ± 0.02	nd
27.707	1824	1838	Neophytadiene	nd	0.2 ± 0.01	nd
27.881	1833	1846	Hexahydrofarnesyl acetone	nd	0.3 ± 0.02	nd
27.981	1838	1861	Myristic acid, TMS derivative	nd	0.2 ± 0.01	nd
29.479	1911	1925	Hexadecanoic acid, methyl ester	nd	0.5 ± 0.02	nd
30.111	1944	1980	Gerany-p-cymene	17.0 ± 0.12	2.6 ± 0.02	9.0 ± 0.02
30.792	1979	1978	Hexadecanoic acid, ethyl ester	nd	0.4 ± 0.02	nd
31.822	2033	2054	Palmitic Acid, TMS derivative	nd	9.5 ± 0.02	9.1 ± 0.02
32.686	2079	2098	(Z,Z)-9,12-Octadecadienoic acid, methyl ester	nd	0.4 ± 0.02	nd
32.809	2086	2101	(Z,Z,Z)-9,12,15-Octadecatrienoic acid, methyl ester	nd	0.6 ± 0.02	nd
33.595	2129	2149	Heptadecanoic acid, TMS derivative	nd	0.2 ± 0.02	nd
33.888	2146	2155	Linoleic acid ethyl ester	nd	0.4 ± 0.01	nd
34.010	2152	2165	(Z,Z,Z)-9,12,15-Octadecatrienoic acid, ethyl ester	nd	0.3 ± 0.00	nd
34.180	2162	2162	Phytol, TMS derivative	nd	2.3 ± 0.03	6.8 ± 0.03
34.816	2197	2212	(Z,Z)-9,12-Octadecadienoic acid, TMS derivative	nd	13.8 ± 0.03	11.6 ± 0.02
34.913	2203	2218	α-Linolenic acid, TMS derivative	nd	16.1 ± 0.02	15.4 ± 0.03
35.322	2227	2250	Stearic acid, TMS derivative	nd	2.1 ± 0.02	1.7 ± 0.02
37.154	2335	2364	4,8,12,16-Tetramethylheptadecan-4-olide	nd	0.6 ± 0.03	nd
38.568	2420	2447	Arachidic acid, TMS derivative	nd	0.7 ± 0.03	nd
39.477	2471	2500	Pentacosane	nd	0.4 ± 0.02	nd
42.559	2615	2644	Behenic acid, TMS derivative	nd	0.5 ± 0.02	nd
44.005	2700	2700	Heptacosane	nd	2.0 ± 0.02	nd
47.185	2761	2800	Octacosane	nd	0.5 ± 0.01	nd
51.296	2860	2900	Nonacosane	17.4 ± 0.05	7.9 ± 0.02	5.5 ± 0.02
56.577	3000	3000	Triacontane	nd	0.9 ± 0.02	nd
63.604	3065	3100	Hentriacontane	nd	9.7 ± 0.02	14.6 ± 0.02
84.108	3264	3300	Tritriacontane	nd	2.5 ± 0.03	nd
			Total identified compounds (%) *	**100.0**	**89.8**	**100.0**

* The total represents the relative percentage of all identified compounds. The remaining percentage corresponds to unidentified peaks that could not be assigned using the available commercial GC–MS libraries. ^1^ RRI: Relative retention index calculated using the n-alkane series. ^2^ RRI Lit.: Relative retention index reported in the literature. Note: Free nerolidol and its O-TMS derivative were integrated as separate chromatographic peaks and are reported separately in the table. The values of 45.4%, 2.1%, and 10.3% refer to the free nerolidol peak. nd: not detected under the applied analytical conditions.

**Table 5:** Compounds identified by GC–MS and relatively quantified by GC–FID in the methanol extract of *Stachys pinardii*. Methanol 1: plantlets cultured on MS medium supplemented with BAP (1.0 mg/L) and IAA (2.0 mg/L); Methanol 2: plantlets cultured on MS medium supplemented with BAP (2.0 mg/L) and IAA (2.0 mg/L); Methanol 3: plants collected from the wild. Values are expressed as mean relative percentages (%) ± SD from three repeated GC–FID analyses of the same extract (*n* = 3).

RT (min)	RRI ^1^	RRI Lit. ^2^	Compounds	Met-1 (%)	Met-2 (%)	Met-3 (%)
13.121	1221	1221	L-Valine, 2TMS derivative	nd	nd	0.2 ± 0.02
13.809	1246	1244	Benzoic acid, TMS derivative	1.6 ± 0.02	10.4 ± 0.02	1.6 ± 0.03
14.803	1282	1296	Glycerol, 3TMS derivative	2.6 ± 0.02	14.0 ± 0.03	2.9 ± 0.01
15.041	1290	1290	Silanol, trimethyl-, phosphate (3:1)	0.3 ± 0.02	nd	0.5 ± 0.02
15.176	1295	1295	Benzeneacetic acid, TMS derivative	0.2 ± 0.01	nd	nd
15.702	1315	1323	Butanedioic acid, 2TMS derivative	0.4 ± 0.02	nd	0.9 ± 0.01
16.056	1328		Carvacrol, TMS derivative	0.7 ± 0.02	nd	1.0 ± 0.02
16.316	1338	1346	Glyceric acid, 3TMS derivative	0.1 ± 0.01	nd	0.1 ± 0.02
16.420	1330	1331	Uracil, 2TMS derivative	nd	nd	0.1 ± 0.02
16.547	1347	1353	(E)-2-Butenedioic acid, 2TMS derivative	0.1 ± 0.02	nd	0.1 ± 0.01
16.774	1356	1355	Nonanoic acid, TMS derivative	0.2 ± 0.02	nd	0.1 ± 0.01
16.984	1364	1369	4-Phenyl-2-butanol, TMS derivative	0.6 ± 0.03	nd	0.1 ± 0.01
17.540	1385	1399	3-phenyl-2-Propenoic acid, methyl ester	nd	nd	0.2 ± 0.02
18.033	1404	1410	Pentanedioic acid, 2TMS derivative	0.1 ± 0.02	nd	0.1 ± 0.01
18.241	1412	1414	Benzenepropanoic acid, TMS derivative	0.1 ± 0.02	nd	nd
19.852	1477	1486	α-Curcumene	0.1 ± 0.01	nd	nd
20.348	1496	1487	Malic acid, 3TMS derivative	0.6 ± 0.03	nd	1.7 ± 0.01
20.590	1506	1510	Hexanedioic acid, 2TMS derivative	5.0 ± 0.03	nd	5.6 ± 0.01
20.682	1510	1524	Salicylic acid, 2TMS derivative	0.3 ± 0.02	nd	nd
20.940	1521	1528	meso-Erythritol, 4TMS derivative	0.4 ± 0.03	nd	0.3 ± 0.02
21.047	1526	1520	L-5-Oxoproline, 2TMS derivative	0.2 ± 0.01	nd	0.4 ± 0.02
21.168	1531	1541	4-Aminobutanoic acid, 3TMS derivative	nd	nd	0.3 ± 0.02
21.384	1540	1540	Cinnamic acid, TMS derivative	0.5 ± 0.02	nd	1.3 ± 0.02
21.797	1558	1564	Nerolidol	0.9 ± 0.03	nd	nd
21.876	1561	1558	1-Dodecanol, TMS derivative	nd	nd	0.1 ± 0.02
22.055	1569	1566	Tyrosol, 2TMS derivative	0.3 ± 0.03	nd	0.1 ± 0.02
23.141	1616	1665	Tartaric acid, 4TMS derivative	nd	nd	0.1 ± 0.01
23.344	1625	1637	4-Hydroxybenzoic acid, 2TMS derivative	0.1 ± 0.02	nd	0.2 ± 0.02
23.786	1644	1657	Dodecanoic acid, TMS derivative	0.2 ± 0.03	nd	nd
23.789	1644	1657	L-Rhamnose, 4TMS derivative	nd	nd	0.1 ± 0.02
24.189	1662	1658	β-D-(-)-Ribopyranose, 4TMS derivative	0.1 ± 0.01	nd	0.2 ± 0.02
24.517	1676		Nerolidol, O-TMS	2.5 ± 0.02	nd	0.8 ± 0.02
24.953	1695	1710	Suberic acid, 2TMS derivative	0.2 ± 0.02	nd	0.3 ± 0.02
25.532	1722	1738	D-Xylose, 4TMS derivative/D-Galactose, 5TMS derivative	nd	nd	0.2 ± 0.02
25.880	1738	1737	D-(+)-Arabitol, 5TMS derivative	1.1 ± 0.02	nd	0.5 ± 0.02
26.419	1763	1774	Vanillic acid, 2TMS derivative	0.3 ± 0.03	nd	0.4 ± 0.02
27.049	1792	1807	Azelaic acid, 2TMS derivative	0.9 ± 0.01	nd	0.6 ± 0.03
27.498	1814	1822	D-(+)-Talofuranose, pentakis(trimethylsilyl) ether (isomer 1)	nd	nd	1.1 ± 0.01
27.689	1823	1813	D-(-)-Tagatofuranose, pentakis(trimethylsilyl) ether (isomer 1)	2.6 ± 0.03	8.7 ± 0.02	3.4 ± 0.02
27.842	1831	1852	D-Allofuranose, pentakis(trimethylsilyl) ether	1.2 ± 0.02	nd	0.7 ± 0.02
27.855	1831	1840	D-(+)-Talofuranose, pentakis(trimethylsilyl) ether (isomer 2)	nd	nd	2.0 ± 0.02
27.915	1834	1829	β-D-Allopyranose, 5TMS derivative	0.6 ± 0.01	nd	0.9 ± 0.01
27.964	1837	1843	Myristic acid, TMS derivative	0.2 ± 0.02	nd	nd
28.045	1841	1840	D-(+)-Talofuranose, pentakis(trimethylsilyl) ether (isomer 2)	0.2 ± 0.03	nd	nd
28.529	1864	1852	1,2,3,5,6-pentakis-O-(trimethylsilyl)-β-D-Galactofuranose	nd	nd	1.8 ± 0.02
28.806	1878	1889	D-(+)-Galactopyranose, 5TMS derivative (isomer 2)	0.8 ± 0.03	nd	1.6 ± 0.02
29.010	1888	1895	Sebacic acid, 2TMS derivative	0.1 ± 0.02	nd	nd
29.275	1901	1885	d-Mannose, pentakis-O-(TMS)-, o-methyloxyme, (1E)-	0.6 ± 0.02	nd	nd
29.287	1902	1897	(1E)-d-Galactose, pentakis-O-(TMS)-, o-methyloxyme	nd	nd	1.1 ± 0.02
29.441	1909	1890	β-D-(+)-Mannopyranose, 5TMS derivative	2.5 ± 0.02	nd	5.1 ± 0.01
29.455	1910	1924	α-D-Glucopyranose, 5TMS derivative	2.5 ± 0.01	10.7 ± 0.02	2.8 ± 0.02
30.114	1944	1980	Gerany-p-cymene	0.6 ± 0.02	nd	nd
30.220	1949	1975	D-Mannitol, 6TMS derivative	0.9 ± 0.02	nd	0.4 ± 0.02
31.250	2002	1971	β-D-Glucopyranose, 5TMS derivative	3.7 ± 0.02	14.8 ± 0.01	5.8 ± 0.02
31.608	2021	2030	Palmitelaidic acid, TMS derivative	1.9 ± 0.01	nd	nd
31.821	2033	2053	Palmitic acid, TMS derivative	6.7 ± 0.02	18.6 ± 0.02	2.7 ± 0.03
32.674	2079	2079	Linoleic acid, methyl ester	0.2 ± 0.03	nd	nd
32.796	2085	2098	Linolenic acid, methyl ester	0.4 ± 0.02	nd	nd
33.173	2106	2073	Myo-Inositol, 6TMS derivative	0.4 ± 0.02	nd	1.0 ± 0.02
33.581	2128	2149	Heptadecanoic acid, TMS derivative	0.2 ± 0.03	nd	nd
33.771	2139	2165	1-Octadecanol, TMS derivative	0.1 ± 0.01	nd	nd
34.167	2163	2183	Phytol, TMS derivative	1.3 ± 0.02	nd	0.7 ± 0.02
34.792	2196	2212	Linoleic acid trimethylsilyl ester	4.2 ± 0.02	9.8 ± 0.02	1.3 ± 0.03
34.924	2204	2218	α-Linolenic acid, trimethylsilyl ester	5.8 ± 0.02	12.9 ± 0.08	2.3 ± 0.02
35.315	2226	2250	Stearic acid, TMS derivative	4.1 ± 0.01	nd	0.8 ± 0.02
37.254	2341	2376	Glyceryl-glycoside TMS ether	nd	nd	0.3 ± 0.03
37.844	2377	2385	Dehydroabietic acid, TMS derivative	nd	nd	0.3 ± 0.01
38.556	2419	2447	Arachidic acid, TMS derivative	0.6 ± 0.02	nd	0.3 ± 0.02
39.157	2453	2469	Uridine, 3TMS derivative	nd	nd	0.2 ± 0.01
39.750	2486	2500	2’,4’,6’-trihydroxy-Chalcone, tris-TMS	0.6 ± 0.02	nd	0.3 ± 0.02
41.662	2577	2574	1-Monopalmitin, 2TMS derivative	0.6 ± 0.01	nd	nd
42.555	2615	2644	Behenic acid, TMS derivative	0.5 ± 0.02	nd	0.2 ± 0.02
44.197	2674		Sucrose, 8TMS derivative	0.3 ± 0.02	nd	1.4 ± 0.02
			Total identified compounds (%) *	**65.1**	**100.0**	**59.6**

* The total represents the relative percentage of all identified compounds. The remaining percentage corresponds to unidentified peaks that could not be assigned using the available commercial GC–MS libraries. Notes: Free nerolidol and its O-TMS derivative were integrated as separate chromatographic peaks and are reported separately in the table. ^1^ RRI: Relative retention index calculated using the n-alkane series. ^2^ RRI Lit.: Relative retention index reported in the literature. nd: not detected under the applied analytical conditions.

## Data Availability

The data presented in this study are available from the corresponding author upon reasonable request.

## Abbreviations

The following abbreviations are used in this manuscript:

BAP	6-Benzylaminopurine
IAA	Indole-3-acetic acid
NAA	Naphthaleneacetic acid
KIN	Kinetin
IBA	Indole-3-butyric acid
GC–MS	Gas chromatography–mass spectrometry
GC–FID	Gas chromatography–flame ionization detection
MS	Murashige and Skoog
